# Efficacy and Characteristics of the Stimuli of Action Observation Therapy in Subjects With Parkinson's Disease: A Systematic Review

**DOI:** 10.3389/fneur.2020.00808

**Published:** 2020-08-13

**Authors:** Federico Temporiti, Paola Adamo, Emanuele Cavalli, Roberto Gatti

**Affiliations:** ^1^Physiotherapy Unit, Humanitas Clinical and Research Center—IRCCS, Milan, Italy; ^2^Department of Biomedical Sciences, Humanitas University, Milan, Italy

**Keywords:** Parkinson's disease, action observation therapy, rehabilitation, functional recovery, motor function

## Abstract

**Background:** The discovery of the Mirror Neuron System has promoted the development of Action Observation Therapy (AOT) to improve motor and functional abilities in patients with Parkinson's disease (PD). This innovative approach involves observing video-clips showing motor contents, which may vary across the studies influencing AOT efficacy. To date, no studies have systematically summarized the effects of AOT in patients with PD on motor and functional outcomes, underlining the characteristics of visual stimuli in relation to their efficacy.

**Objectives:** To describe the potential benefits of AOT in patients with PD and discuss the characteristics of visual stimuli used in clinical studies in relation to their efficacy.

**Methods:** A systematic literature search was carried out using MEDLINE via PubMed, EMBASE, Scopus, and PEDro, from inception until March 2020. Randomized controlled trials that investigated the effects of AOT on motor and functional recovery in patients with PD were included. Two independent reviewers appraised the records for inclusion, assessed the methodological quality, and extracted the following data: number and characteristics of participants, features and posology of the treatments, outcome measures at each follow-up, and main results. Findings were aggregated into a quantitative synthesis (mean difference and 95% confidence interval) for each time point.

**Results:** Overall, 7 studies (189 participants) with a mean PEDro score of 6.1 (range: 4–8) points were selected. Included studies revealed AOT as effective in improving walking ability and typical motor signs (i.e., freezing of gait and bradykinesia) in patients with PD. Moreover, when this approach incorporated ecological auditory stimuli, changes to functional abilities and quality of life were also induced, which persisted up to 3 months after treatment. However, included studies adopted AOT stimuli with heterogeneous posology (from a single session to 8 weeks) and characteristics of motor contents might be responsible for different motor and functional recovery (person-related and viewing perspectives, transitive or intransitive actions, healthy subjects or patients, and association or not with imitation).

**Conclusions:** AOT leads to improvements in motor and functional abilities in patients with PD and the characteristics of visual stimuli may play a role in determining AOT effects, deserving further investigations.

## Introduction

Parkinson's disease (PD) represents a progressive neurodegenerative disorder affecting about 6 million adults worldwide with greater incidence over 60 years of age (1–3). Motor manifestations (i.e., tremor, bradykinesia, muscular rigidity, postural instability, and abnormal gait patterns) and non-motor signs and symptoms (i.e., cognitive and autonomic dysfunctions, sleep disorders, fatigue, and depression) are common deficits causing disability, with consequences on participation and quality of life (4). In addition to pharmacological and surgical interventions, rehabilitation of motor function represents an effective tool to alleviate motor manifestations related to this condition (5–7). Rehabilitation in PD patients consists of approaches addressed to enhance functional abilities in order to reduce disability, improve quality of life, and minimize secondary complications of the disease (8, 9). The most common rehabilitative interventions include physical exercise (i.e., aerobic, resistance, and balance training as well as mobility and coordination exercises), walking training, and other activities such as dance or martial arts, which are often practiced in association with cues (8).

In this scenario, the discovery of the Mirror Neuron System (MNS) has promoted the development of Action Observation Therapy (AOT), which represents an innovative rehabilitative approach involving action observation with or without motor imagery and imitation of observed tasks (10–12). This approach takes advantage of the peculiarity of the Mirror Neurons System, which shows an activity during both execution and observation of actions, playing a key role in understanding actions performed by others (13). These neurons also discharge during the internal rehearsal of motor actions (motor imagery) and are implicated in motor learning through the building of a motor memory (14, 15). In particular, motor memory is a process that enables humans to plan, select, learn, and recall motor behaviors thanks to the interaction between pre-existing and new motor programs (16, 17). Neurophysiological findings have described MNS as an operating cerebral network in PD patients, able to play a potential compensatory role on brain functional alterations responsible for motor deficits (12). Consequently, studies aimed at investigating the effects of AOT on motor and functional abilities have been published over the past years, suggesting that AOT improves autonomy, walking ability, or typical motor signs such as freezing of gait and bradykinesia in patients with PD (17–20); however, a systematic review on this topic is missing. In particular, a single meta-analysis investigating the effectiveness of physiotherapy in these patients have reported positive results of AOT on freezing of gait (21), but the efficacy of this innovative tool on other functional outcome measures adopted in rehabilitation of patients with PD has never been systematically quantified. Moreover, AOT can be delivered alone or in association with usual physiotherapy through video-clips representing motor contents (20, 22). However, characteristics and motor contents of the stimuli delivered to patients vary across the studies (i.e., first-person or/and third-person, transitive or/and intransitive actions, healthy subjects or patients with the same condition as the viewers) (11, 23) and the efficacy of AOT could depend on the characteristics of the visual stimuli delivered to patients in reference to their motor impairment. Additionally, identification of the most appropriate AOT features may enhance the recruitment of the MNS, augmenting motor learning induced by this approach (11). However, to date, no studies have underlined the characteristics of AOT stimuli used in clinical trials in relation to their efficacy.

Against this background, it is relevant to conduct a systematic research aimed at pointing out the efficacy of AOT in patients with PD on motor and functional recovery and discussing the features of visual stimuli used in clinical studies, in order to underline the most effective stimuli.

## Materials and Methods

This systematic review was conducted in accordance with the guidelines outlined by Preferred Reporting Items for Systematic Reviews and Meta-analysis (PRISMA) statement (24).

### Data Sources and Search Strategy

A literature search was carried out using the academic databases MEDLINE via PubMed, EMBASE, Scopus, and PEDro, from inception until March 2020. The search strategy included terms related to “Parkinson's disease,” “action observation,” “action observation therapy,” “action observation training,” and synonymous expressions, which were searched as keywords and free words in titles and abstracts in all databases. The extended version of the PubMed search strategy is provided in Appendix A (Supplementary Material). The reference lists of articles of interest were manually checked in order to find additional relevant studies.

### Eligibility Criteria

The studies meeting the following inclusion criteria were included in the current review: (1) participants with clinical diagnosis of PD according to UK Parkinson's Disease Society Brain Bank criteria (25); (2) randomized controlled trials on rehabilitative intervention focused on AOT with no restrictions on duration, frequency, and characteristics of the stimuli; (3) comparison with any kind of intervention or placebo or no intervention; (4) outcomes related to motor and/or functional recovery assessed at any time point through clinical or instrumental tools; (5) studies written in English. No restrictions on age, disease duration, and severity of the condition were adopted. Overlapping or duplicated articles, thesis and conference proceedings, and abstracts were excluded.

### Study Selection

Two independent reviewers carried out the literature search and all results were imported into EndNote X9 for screening. First, titles, and abstracts were screened to identify relevant studies; subsequently, the full text of the studies retained during the previous step was screened by the two reviewers, independently. In case of disagreement, a third reviewer facilitated the decision process.

### Risk of Bias Assessment

Two independent reviewers assessed risk of bias of included studies through the PEDro scale. It represents an effective tool to evaluate methodological quality of clinical trials in rehabilitation; it is composed of 11 items that can contribute 1 point to the total score (10 points), except for item 1 (eligibility criteria), which is dichotomous (yes/no). Articles with a score ≥ 6 were considered as high quality, those with scores of 5 or 4 were considered as fair quality, and those with a score ≤ 3 were defined as low quality (26). In case of disagreement between the two reviewers during the rating process, a third reviewer was consulted to achieve a consensus.

### Data Extraction and Synthesis

A reviewer extracted details of included studies (number and characteristics of p articipants, features, and posology of the treatments, outcome measures, and significant main findings). A second reviewer checked the correctness of the data extraction process and any disagreements were resolved through consultation with a third reviewer. Findings of eligible studies were aggregated into a quantitative synthesis and presented as tables. In particular, results of single studies were presented for outcomes measure at baseline and follow-up as mean difference and 95% confidence interval. The analysis was performed through the software RevMan 5.3 from the Cochrane Library.

## Results

Table 1 shows the characteristics of the included articles.

**Table 1:** Characteristics of included studies.

**Study**	**Participants**	**AOT group intervention**	**Control group intervention**	**Posology of interventions**	**Characteristics of AOT stimuli**	**Clinical and instrumental outcomes**
Agosta et al. (27)	**25 PD**: item 3 FoG-Q ≥ 2; DD ≥ 5 y, H&Y <4, MMSE > 24. **AOT group**: *n* = 12, 69 ± 8 y, M/F 10/2. **Control group**: *n* = 13, 64 ± 7 y, M/F 8/5.	6 video-clips per week, showing actions with auditory cues associated to movements. After each video-clip, imitation of observed actions at the beat of auditory cues.	Landscape images and execution of the same exercises of AOT group.	**Training**: 12 sessions (3 sessions per week, for 4 weeks). **Each session**: 1 h (24 min of observation and 36 min of execution).	**Motor contents**: body-weight shifting, stepping, walking, turning around a chair, stepping an obstacle, walking through a doorway. •Third-person perspective •Healthy subjects •Frontal viewing perspective	**Clinical**: UPDRS-III (on/off), FoG-Q, UPDRS-II-FoG (on/off), PDQ-39, BBS, 10 MWT. **Time points**: baseline, after 4 weeks of training, at 1 month.
Buccino et al. (20)	**15 PD:** 17–75 y, MMSE > 24. **AOT group**: *n* = 7, 59–80 y, M/F 5/2, DD: 5–19 y. **Control group**: *n* = 8, 67.5–76.5 y, M/F: 5/3, DD: 5.5–13.5 y.	Video-clips showing daily activities plus conventional physiotherapy. Imitation of observed actions.	Video-clips without motor contents plus conventional physiotherapy. Performance of the same actions of the AOT group.	Not specified.	**Motor contents**: functional daily activities.	**Clinical**: UPDRS and FIM. **Time points**: before and after treatment.
Jaywant et al. (19)	**23 PD**, H&Y 1–3, UPDRS gait item ≥ 1. **AOT group**: *n* = 13, 63.7 ± 6.2 y, M/F: 6/7. **Control group**: *n* = 10, 65.8 ± 8.7 y, M/F 4/6.	56 video-clips with PD patients and 56 video-clips with healthy subjects. Participants had to judge whether the observed walking appeared healthy or PD-like gait pattern.	56 video-clips showing water moving roughly and 56 video-clips showing water moving calmly. Participants had to judge whether the water motion was roughly or calmly.	**Training:** 7 days. **Each session**: Not specified.	**Motor contents**: walking in hallway. •Third-person perspective •Healthy and PD subjects •Frontal, lateral, and posterior viewing perspective.	**Clinical**: PDQ-39 mobility. **Instrumental**: Spatial–temporal gait parameters during straight-line walking, walking with turns, and dual-task walking. **Time points**: before and after 8 days of training.
Mezzarobba et al. (28)	**24 PD** with FoG, H&Y: 1–3, BDI ≤ 16, MMSE > 24. **AOT group**: *n* = 12, 74.6 ± 5.9 y M/F: 7/5, DD: 10.7 ± 3.44. **Control group**: *n* = 12, 72 ± 5.87 y, 7/3 M/F, DD: 9.4 ± 4.8.	32 video-clips with 8 gait-related gestures associated to ecological cues. After each video-clip, patients had to practice the same actions for the same amount of time watching the same video-clip.	Execution of the same 8 motor gestures of AOT group through visual or auditory cues. Participants progressively learned to perform gestures without cues.	**Training**: twice a week for 8 weeks **Each session**: 1 h	**Motor contents**: body-weight shifting, taking a step, gait initiation, turn around, stepping over an obstacle, sit-to-walk, normal walking, walking through a doorway. •Third-person perspective •Healthy subjects •Frontal and lateral viewing-perspective	**Clinical**: NFOG-Q, UPDRS-II, UPDRS-III, PDQ-39, TUG, 6 MWT, BBS.**Time points**: baseline, after 8 weeks of training, at 1 and 3 months
Pelosin et al. (29)	**18 PD:** 59–81 y, M/F: 8/12, FOG-Q item 3 ≥ 2 and item 4 ≥ 1), MMSE > 24 **AOT group**: *n* = 9, 68.8 ± 4.1 y, DD: 11.6 ± 4.9 y. **Control group**: *n* = 9, 70.2 ± 6.8 y, DD: 9.5 ± 3.7.	6 video-clips, 6 min each. After observation, patients had to imitate observed actions.	Video-clips showing static landscapes images. After, observation patients had to perform the same movements of AOT group.	**Training** 3 sessions per week, for 4 weeks. **Each session**: 1 h	**Motor contents**: body-weight shifting, stepping, normal walking, turning around a chair, stepping an obstacle, walking through a doorway. •Third-person perspective •Healthy subjects •Frontal viewing perspective	**Clinical**: FOG-Q, FoG-diary, TUG, 10 MWT, Tinetti scale, BBS, and PDQ-39. **Time points**: before training, 2 days, and 4 weeks after training.
Pelosin et al. (18)	**20 PD**: H&Y 1–3, MMSE ≥ 24. **AOT group**: *n* = 10, 68.8 ± 7.4 y, M/F: 3/7, DD: 9.1 ± 3.7. **Control group**: *n* = 10, 66.4 ± 8.9 y; M/F: 4/6, DD: 8.9 ± 3.1 y.	Observation of repetitive finger movements (opposition of thumb to index, medium, ring, and little fingers) paced at 3 Hz.	Listening of acoustic cues paced at 3 Hz.	**Training:** 1 session of 6 min	**Motor contents**: finger opposition with the right hand. •Third-person perspective •Healthy subjects	**Instrumental**: spontaneous movement rate, inter-tapping interval, and touch duration. **Time points**: baseline, immediately after, 45 min, and 2 days after training
Pelosin et al. (17)	**64 PD**: H&Y 2–3, able to walk unassisted; FOG-Q: item 2 ≥ 1 and item 4 ≥ 2, MMSE > 24. **AOT group**: *n* = 33, 70.4 ± 4.5 y, M/F: 16/17, DD: 10.7 ± 3.9 y. **Control group**: *n* = 31, 72.8 ± 3.1 y, M/F: 15/16, DD: 9.5 ± 4.2 y.	6 video-clips, 6 min each. After observation, in the remaining time (36 min) patients had to imitate observed actions.	Video-clips showing static landscapes images. After, observation patients had to perform the same movements of AOT group.	**Training:** 2 times per week for 5 weeks **Each session**: 45 min	**Motor contents**: body-weight shifting, stepping, normal walking, turning around a chair, stepping an obstacle, walking through a doorway. •Third-person perspective •Healthy subjects •Frontal viewing perspective	**Clinical**: FOG-Q, TUG, 10 MWT, and BBS **Time points**: baseline, 1 and 4 weeks after training.

*AOT, Action Observation Therapy; PD, Parkinson's disease; M, male; F, female; y, year; DD, disease duration; FOG-Q, Freezing of Gait Questionnaire; H&Y, Hoehn&Yahr; MMSE, Mini-Mental Status Examination; BDI, Beck Depression Inventory; UPDRS, Unified Parkinson's Disease Rating Scale; PDQ-39, Parkinson's Disease Questionnaire−39 items; 10 MWT, 10 Meters Walking Test; BBS, Berg Balance Scale; FIM, Functional Independence Measure; NFOG-Q, New Freezing of Gait Questionnaire; 6 MWT, 6 Minutes Walking Test; FoG-diary, Freezing of Gait diary*.

### Selection of the Studies

**Figure 1:**
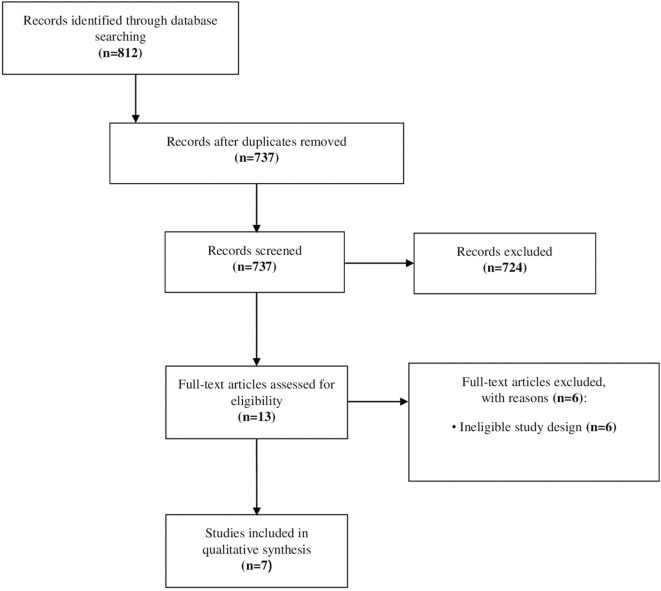
Flowchart of the study selection.

In total, 812 records were identified through literature search procedures. Once duplicates (75 records) had been removed, and titles and abstracts were screened, the full text of 13 articles was evaluated for the final inclusion. Finally, 7 articles were selected for the current review. The selection flow chart is shown in Figure 1.

### Participants

All patients of the studies were able to walk unassisted and had mild to moderate disease severity with a Hoehn&Yahr (H&Y) score of 2 or 3. Studies included participants with a disease duration of at least 5 years and without dementia (Mini-Mental Status Examination > 24). Four studies included patients with freezing of gait, with an incidence of at least one episode in a week and a duration of at least 2 s for each episode (17, 27–29). Finally, in addition to a PD control group, two studies provided a sample of healthy controls matched for age and sex with patients (18, 27).

### Characteristics of AOT Interventions

AOT was administered alone (17–19, 27–29) or in association with conventional physiotherapy (20) using video-clips projected on a laptop (17–20, 27, 28) or on a wall located in front of participants (17). During observation, participants were asked to keep their attention on movement details without performing any kind of movement. Only one study, after observation of each video-clip, asked patients to imitate the observed actions while they were still watching the same video (28). Other four studies asked patients to imitate the observed tasks after observation (17, 20, 27, 29), whereas the remaining two studies delivered AOT without imitation (18, 19). Moreover, Agosta et al. (27) asked participants to follow auditory cues during imitation, whereas in the study of Mezzarobba et al. (28) ecological auditory cues were delivered to patients during AOT. Six studies used video-clips showing healthy actors performing actions. Only Jaywant et al. (19) proposed AOT stimuli representing patients with PD performing walking trials in addition to healthy individuals and asked observers to judge if the observed walking task was performed by healthy or PD actors. Motor contents of AOT stimuli represented activities such as walking in different contexts and gait-related tasks (17, 19, 27–29), functional daily tasks (20) or intransitive upper limb tasks as in finger movements (18). All observed actions were delivered using a third-person perspective and from a frontal (17, 27, 29), frontal and lateral (28), and frontal, lateral, and posterior (19) views. The mean duration of each session of training was 56 min (range, 45–60 min). Specifically, 24 min consisted in observing video-clips, whereas the remaining time was dedicated to imitation of observed actions (17, 27–29). The duration of the treatment period was 1 week (19), 4 weeks (17, 27, 29), or 8 weeks (28). A single study explored the effects of a single session of AOT lasting 6 min (18), whereas Buccino et al. (20) gave no information on the treatment duration.

### Characteristics of Control Interventions

Control groups received the same posology of AOT intervention in terms of frequency and duration in all studies. In four studies, participants of control groups were asked to watch video-clips showing static landscapes without any motor content (17, 20, 27, 29). After observation, patients had to practice the same motor tasks of the AOT group, following the instructions of an operator. In two studies, where AOT was not associated with imitation, control groups observed landscapes with moving water (19) or listened to acoustic cues paced at 3 Hz (18). Finally, in the study of Mezzarobba et al. (28) the control group did not watch any video-clips, but performed motor tasks following auditory or visual cues.

### Outcome Measures

Unified Parkinson's Disease Rating Scale (UPDRS) for disease severity was assessed in three studies (20, 27, 28). Four studies focused on improvement in freezing of gait episodes assessed through the Freezing of Gait Diary (FoG-diary) (29), Freezing of Gait Questionnaire (FoG-Q) (17, 27, 29), or the New Freezing of Gait Questionnaire (NFoG-Q) (28). The Parkinson's Disease Questionnaire−39 items (PDQ-39) was used to assess quality of life (19, 27–29), whereas Berg Balance Scale (BBS) (17, 27–29), Tinetti Scale (29), 10 Meters Walking Test (10 MWT) (17, 27, 29), Timed Up and Go test (TUG) (17, 28, 29), and 6 Minutes Walking Test (6 MWT) (28) and Functional Independence Measure (FIM) (20) were adopted as measures of balance, gait speed, functional mobility, endurance, and autonomy. Moreover, Jaywant et al. (19) analyzed spatial–temporal gait parameters during walking in a straight line, with turns, and during a dual task. Finally, Pelosin et al. (18) assessed spontaneous movement rate, inter-tapping intervals, and touch duration during self-paced finger opposition movements in order to understand the effects of AOT on the spontaneous rate of finger movements (18).

### Methodological Quality

The risk of bias score of the included studies is shown in Appendix B (Supplementary Material). PEDro scores of included studies ranged from 4 to 8 points with an average of 6.1 points. The methodological quality of 4 studies was high (19, 27–29), whereas the other three studies had a moderate quality (17, 18, 20). In particular, all studies did not report blinding of participants and therapists, four studies had no allocation concealment (17, 18, 20, 29) and did not declare intention-to-treat analysis (17, 18, 20, 27), and two studies did not specify the number of missing data at follow-up (18, 20) and blindness of the assessors (17, 19); in another study, there was no reporting of measure of variability (20). Finally, on just one occasion, a PEDro scale item was scored differently by the two reviewers, but after the consultation of the third rater, agreement was reached.

### Efficacy of AOT

Results of the current review suggest the efficacy of AOT on motor and functional outcomes in patients with PD, although disagreement among the authors' results was found in some outcomes (Table 2). AOT effects were found on walking ability (mean difference −2.2 s for 10 MWT) and typical motor signs of the disease as freezing of gait (mean difference from −1.6 to −5.8 for FoG-diary and from −5.7 to −6.3 for NFoG-Q) and bradykinesia (mean difference: −145 ms for inter-tapping interval). Moreover, additional benefits on disability (mean difference: from −5.6 to −7.0 for UPDRS-II and from −17.8 to 23.2 for UPDRS-III) and quality of life (mean difference: from 28.1 to −31.1 for PDQ-39 related to mobility and −18.7 for PDQ-39 related to bodily discomfort) were found when the intervention was associated with ecological auditory cues (17, 18, 28, 29). In particular, when considering walking ability and related disorders, one study found an effect of AOT on 10 MWT 1 week after the training (17). Moreover, AOT reduced incidence of freezing of gait episodes 2 days, 1, 2, 3, and 4 weeks after the training during walking initiation, and 2, 3, and 4 weeks after the training during turn and in terms of total number of episodes (29). A study reported similar findings, demonstrating that 8 weeks of AOT delivered in association with ecological auditory stimuli produced large improvements for NFoG-Q and UPDRS III directly after the intervention and after 1 and 3 months (28). Moreover, this approach revealed also significant effects for UPDRS II and PDQ-39 related to mobility 1 and 3 months after training, and for PDQ-39 related to bodily discomfort dimension directly after the end of the training (28). In addition, despite the lack of follow-up data, Buccino et al. (20) reported a significant improvement in terms of functional independence (FIM) and disability (UPDRS). Finally, when a single session of AOT was applied to reduce bradykinesia during repetitive finger movements, a reduction of interval duration was found for a finger-tapping task, when compared to acoustic cues intervention. These benefits were found 45 min and 2 days after the intervention (18).

**Table 2:** Results of included studies with outcomes presented as mean difference and 95% confidence interval (95% CI) comparing Action Observation Therapy (AOT) with control interventions.

**Outcome measures**		**Time point**	**Mean difference [95% CI]**
**Agosta et al**. **(****27****)**			
*Action Observation Training (Group 1) vs. Landscape Observation Training (Group 2)*			
UPDRS-III off		Post-training	1.20 [−6.89, 9.29]
UPDRS-III on		Post-training	−1.10 [−7.55, 5.35]
		4 wk	1.20 [−6.55, 8.95]
FoG-Q		Post-training	−1.20 [−3.79, 1.39]
		4 wk	−1.10 [−3.31, 1.11]
UPDRS-II-FoG off		Post-training	−0.28 [−0.98, 0.42]
		4 wk	0.13 [−0.73, 0.99]
UPDRS-II-FoG on		Post-training	−0.07 [−0.73, 0.59]
		4 wk	−0.03 [−0.80, 0.74]
PDQ-39		Post-training	−0.07 [−0.73, 0.59]
		4 wk	−0.03 [−0.80, 0.74]
BBS		Post-training	−0.80 [−2.82, 1.22]
		4 wk	−1.00 [−3.06, 1.06]
10 MWT normal speed (s)		Post-training	**1.00 [0.08, 1.92]**
		4 wk	0.52 [−0.75, 1.79]
10 MWT maximum speed (s)		Post-training	0.40 [−0.59, 1.39]
		4 wk	0.00 [−1.51, 1.51]
**Buccino et al**. **(****20****)**			
*Action Observation Training (Group 1) vs. Non-motor Observation Training (Group 2)*			
UPDRS and FIM		Before training	Not available
		Post-training	Not available
**Jaywant et al**. **(****19****)**			
*Action Observation Training (Group 1) vs. Landscape Observation Training (Group 2)*			
Walking	Walking speed (m/s)	1 wk	0.01 [−0.32, 0.34]
straight-line	Stride length (m)	1 wk	0.01 [−0.46, 0.48]
	Stride frequency (strides/s)	1 wk	0.00 [−0.17, 0.17]
	Swing time (% of stride)	1 wk	0.80 [−3.78, 5.38]
	Gait asymmetry	1 wk	0.01 [−0.03, 0.05]
Walking with	Walking speed (m/s)	1 wk	0.00 [−0.30, 0.30]
turns	Stride length (m)	1 wk	0.01 [−0.44, 0.46]
	Stride frequency (strides/s)	1 wk	0.01 [−0.17, 0.19]
	Swing time (% of stride)	1 wk	0.60 [−3.44, 4.64]
	Gait asymmetry	1 wk	0.00 [−0.03, 0.03]
Walking with	Walking speed (m/s)	1 wk	0.00 [−0.46, 0.46]
dual task	Stride length (m)	1 wk	0.00 [−0.53, 0.53]
	Stride frequency (strides/s)	1 wk	0.00 [−0.21, 0.21]
	Swing time (% of stride)	1 wk	0.70 [−4.30, 5.70]
	Gait asymmetry	1 wk	0.00 [−0.07, 0.07]
PDQ-39 mobility		1 wk	−3.10 [−8.83, 2.64]
**Mezzarobba et al**. **(****28****)**			
*Action Observation plus Sonification Training (Group 1) vs. Motor Gesture with Visual and Auditory Cues (Group 2)*			
NFoG-Q		Post-training	**−5.74 [−11.27**, **−0.22]**
		1 mo	**−6.03 [−11.56**, **−0.50]**
		3 mo	**−6.28 [−11.81**, **−0.76]**
UPDRS-II		Post-training	−4.39 [−9.64, 0.86]
		1 mo	**−5.63 [−10.88**, **−0.38]**
		3 mo	–**7.03 [−12.28**, **−1.78]**
UPDRS-III		Post-training	**−23.19 [−33.15**, **−13.22]**
		1 mo	**−14.84 [−24.81**, **−4.87]**
		3 mo	**−17.79 [−27.76**, **−7.83]**
PDQ-39 mobility		Post-training	−14.68 [−35.17, 5.81]
		1 mo	**−28.13 [−48.62**, **−7.64]**
		3 mo	**−31.15 [−51.64**, **−10.67]**
PDQ-39 bodily discomfort		Post-training	**−18.66 [−35.87**, **−1.44]**
		1 mo	−10.14 [−27.35, 7.08]
		3 mo	−13.05 [−30.27, 4.16]
PDQ-39 total		Post-training	−7.89 [−31.65, 15.87]
		1 mo	−23.19 [−46.95, 0.56]
		3 mo	−21.21 [−44.97, 2.55]
TUG (s), 6 MWT (s) and BBS		Post-training	Not significant
		1 mo	Not significant
		3 mo	Not significant
**Pelosin et al**. **(****29****)**			
*Action Observation Training (Group 1) vs. Landscape Observation Training (Group 2)*			
FoG-Q		2 days	−1.60 [−3.40, 0.20]
		4 wk	−2.30 [−4.75, 0.15]
FoG-diary (number of episodes) during start walking		2 days	**−2.10 [−3.70**, **−0.50]**
		1 wk	**−1.89 [−3.63**, **−0.14]**
		2 wk	**−2.84 [−4.81**, **−0.88]**
		3 wk	**−3.77 [−5.39**, **−2.16]**
		4 wk	**−4.04 [−5.86**, **−2.22]**
FoG-diary (number of episodes) during turn		2 days	**−2.20 [−3.81**, **−0.59]**
		1 wk	−1.17 [−2.53, 0.19]
		2 wk	**−3.01 [−4.42**, **−1.60]**
		3 wk	**−4.73 [−6.16**, **−3.30]**
		4 wk	**−5.81 [−7.38**, **−4.23]**
FoG-diary (number of episodes) during obstacle negotiation		2 days	0.36 [−0.64, 1.36]
		1 wk	0.38 [−0.50, 1.25]
		2 wk	−0.32 [−1.63, 0.98]
		3 wk	−0.36 [−1.71, 0.99]
		4 wk	−0.61 [−1.92, 0.69]
FoG-diary (total number of episodes)		2 days	−0.91 [−2.28, 0.47]
		1 wk	−0.58 [−1.83, 0.68]
		2 wk	**−1.63 [−2.99**, **−0.27]**
		3 wk	**−2.47 [−3.85**, **−1.08]**
		4 wk	**−3.15 [−4.58**, **−1.73]**
TUG (s), 10 MWT (s), Tinetti Scale, BBS, and PDQ-39		2 days	Not significant
		1 wk	Not significant
		2 wk	Not significant
		3 wk	Not significant
		4 wk	Not significant
**Pelosin et al**. **(****18****)**			
*Action Observation Training (Group 1) vs. Acoustic Training (Group 2)*			
Self-paced movement rate (Hz)		Immediately post-training	0.04 [−0.40, 0.47]
		45 min	0.31 [−0.21, 0.83]
		2 days	0.36 [−0.03, 0.75]
Inter tapping interval (ms)		Immediately post-training	−59.62 [−130.01, 10.77]
		45 min	**−140.81 [−200.58**, **−81.04]**
		2 days	**−145.87 [−211.12**, **−80.62]**
Touch duration (ms)		Immediately post-training	55.39 [−118.84, 229.62]
		45 min	59.61 [−129.00, 248.22]
		2 days	25.85 [−162.70, 214.40]
**Pelosin et al**. **(****17****)**			
*Action Observation Training (Group1) vs. Landscape Observation Training (Group2)*			
FoG-Q		1 wk	−0.80 [−3.47, 1.87]
		4 wk	−2.60 [−5.46, 0.26]
TUG (s)		1 wk	−1.20 [−3.98, 1.58]
		4 wk	−2.60 [−5.43, 0.23]
BBS		1 wk	−1.10 [−3.67, 1.47]
		4 wk	1.90 [−0.91, 4.71]
10 MWT (s)		1 wk	**−2.20 [−4.26**, **−0.14]**
		4 wk	−1.60 [−4.05, 0.85]

*FOG-Q, Freezing of Gait Questionnaire; UPDRS, Unified Parkinson's Disease Rating Scale; PDQ-39, Parkinson's Disease Questionnaire -39 items; 10 MWT, 10 Meters Walking Test; BBS, Berg Balance Scale; FIM, Functional Independence Measure; NFOG-Q, New Freezing of Gait Questionnaire; 6 MWT, 6 Minutes Walking Test; FoG-diary, Freezing of Gait diary; TUG, Timed Up and Go test; wk, week; mo, month*.

*Significant results are reported in bold*.

## Discussion

The aim of the review was to summarize the effects of AOT in patients with PD and discuss the features of visual stimuli used in clinical studies in relation to their efficacy. Seven RCTs including 189 participants focused on AOT effects on walking ability, typical motor signs, such as freezing of gait and bradykinesia, balance, functional mobility, endurance, disability in daily activities, and quality of life, matched the inclusion criteria. Participants of included studies satisfied the UK Parkinson's Disease Society Brain Bank criteria and were reported as outpatients, except for the study of Buccino et al. where they were inpatients of a hospital rehabilitation department. Patients had mild to moderate disease severity (H&Y 2–3), no dementia, and a disease duration >5 years.

### AOT Efficacy

Five studies suggested AOT as an effective approach to improve walking ability and typical motor signs (i.e., freezing of gait and bradykinesia) in patients with PD. Moreover, when AOT incorporated ecological auditory stimuli, additional improvements were shown in terms of disability (up to 3 months after the end of the training) and quality of life related to mobility (1 and 3 months after the training) and bodily discomfort (directly after the training) (28). A single study reported improvements in autonomy in hospitalized patients (20). Interestingly, the neural underpinnings of AOT in patients with PD seem to imply the ability of this approach to induce a functional reorganization of the circuits connecting the motor cortex with basal ganglia and the projections from motor cortex to thalamus (30, 31).

Walking represents one of the most compromised daily activities in patients with PD, where the occurrence of typical phenomenon such as freezing of gait increases risk of fall, affecting social participation and quality of life (32, 33). In this review, two studies described the efficacy of AOT on daily frequency of freezing of gait (28, 29). Pelosin et al. (29) demonstrated that a reduction of this frequency took place especially during step initiation and turning phases of gait, circumstances that imply an increase in attentional load. In fact, freezing of gait seems to be triggered by both motor and cognitive factors, which can be improved through the building of a motor memory induced by the observation of actions followed by their imitation (34). Neurophysiological studies have suggested a decrease in supplementary motor area activity, compensated by increased recruitment of basal ganglia during walking in patients with PD (35). When this subcortical hyperactivity collapses in presence of events that require changes in motor planning, the phenomenon of freezing of gait occurs (35). Surprisingly, AOT intervention enhances the recruitment of areas involved in the MNS (premotor cortex, inferior frontal gyrus, and left inferior parietal lobule) as well as fronto-parietal areas (left superior/inferior parietal and right precentral gyri) responsible for attentive processes in response to sudden environmental changes, allowing for reduction in freezing of gait frequency (27, 29). In addition, Mezzarobba et al. (28) demonstrated that when a congruent multisensory stimulation was associated with AOT, effects were amplified, probably thanks to a facilitation in mental representation of observed tasks due to a reduction in cognitive load (36). In this circumstance, benefits were also extended to disability (UPDRS-II and UPDRS-III) and quality of life (PDQ-39) (28). In fact, fMRI studies have demonstrated that observation of actions in association with congruent auditory stimuli increases the activity in superior and medial posterior temporal regions as well as in the insula and the right precentral gyrus, and reinforces the functional connectivity between basal ganglia and frontal and parietal cortical motor areas (36). These regions belong to MNS and cover a key role in sensory integration and cognitive processes (36). Similarly, although Agosta et al. (27) found no differences between experimental and control groups in clinical outcomes, within-group improvements for gait ability and quality of life in AOT group were associated with increased recruitment of fronto-parietal network during observation and execution of a motor task in fMRI. Positive results of AOT have also been documented for the upper limb, where the observation of finger movements seems to increase the finger tapping rate in both healthy subjects and patients with PD. Also in this case, observation of the same task before its performance has been hypothesized to influence the retention of motor information, improving the temporal organization of movements (18).

The number of participants in the included studies was relatively small (from 15 to 25 patients), except for the study of Pelosin et al. (17) (64 patients), and none of the studies estimated sample size a priori. Moreover, not all studies scored well for methodological quality, in particular three studies, which revealed a PEDro score lower than 6 points (moderate quality) (26). In these studies, blinding of participants and assessors were not applied and the lack of concealed allocation and intention-to-treat analysis might overestimate the effects of the treatment. In addition, no homogeneity in terms of AOT frequency and duration of the treatments was adopted among the included studies, with potential consequences on AOT effects and their persistence over time. In fact, it is reasonable to speculate that the duration of treatment for only 7 days as adopted by Jaywant et al. (19) might not be enough to produce detectable changes on motor abilities. On the other hand, as reported by Mezzarobba et al. (28) 8 weeks of treatment might have contributed to the size of observed benefits. The frequency, ranging from 2 to 3 sessions per week, matched with that suggested by literature in order to maximize the retention of the acquired motor skills (37, 38). The only exceptions were the studies by Jaywant et al. (19) which applied AOT every day, and Pelosin et al. (18), where effects induced by a single session of AOT were investigated (18). Moreover, although walking ability and freezing of gait represented the most assessed variables, a considerable heterogeneity of outcomes was detected, and limitations of some outcome measures must be acknowledged. This is the case of NFoG-Q, where a modest reliability and poor responsiveness with a high Minimal Detectable Change has been described in these patients (39). Finally, only two studies included the assessment of disability and quality of life in addition to patients' motor impairment.

### Characteristics of the Stimuli

The characteristics of AOT stimuli vary across the included studies with some research suggesting an association between features of video-clips and AOT efficacy (11). Studies have described additional benefits when AOT is associated with motor imagery in both healthy subjects and patients with neurological disorders (40–43). However, none of the studies administering AOT in subjects with PD took into account the association between AOT and motor imagery. It is worth noting that motor imagery ability in these patients seems to be preserved, especially in early stages, supporting the possible use of this approach as adjuvant to other rehabilitative interventions (12).

AOT is delivered using video-clips representing subjects that execute motor tasks, and its effects on motor recovery may also depend on a person-related perspective from which actions are observed (i.e., first- or third-person perspective and specular or anatomical view in case of first-person perspective) (23, 44, 45). Perspective influences elicited not only brain activity but also the ability to imitate, and higher involvement of a sensorimotor pattern and simplicity in imitation of actions observed in first person was described, when compared to a third-person perspective (44, 46). Moreover, first-person perspective seems to enhance kinesthetic perception, more than third-person perspective, enabling the vividness of mental representation, and improving the imitation of the observed actions (45–48). When investigating AOT applications in neurorehabilitation, this approach is delivered in both perspectives, and studies reporting results after AOT in first-person perspective focused on upper limb rehabilitation (49–51). In the studies considered for the review, given that they were AOT interventions with the focus on improving walking or balance abilities and functional independence in daily activities, the stimuli were delivered from a third-person perspective. Moreover, a third-person perspective was adopted to improve upper limb bradykinesia (18). To date, a single pilot study, which was not included in our review due to the lack of random allocation, explored the feasibility of AOT delivered from a first-person perspective to improve balance and mobility in patients with PD, revealing potential benefits (52).

It is worth underlining that MNS activity during AOT also seems to be influenced by empathy of observers, which is the ability to understand and perceive what another person is experiencing (53, 54). Additionally, studies reported that, not only person-related perspective, but also viewing perspective represent a potential influencing factor on AOT efficacy (55). In particular, observing actions from a perspective that emphasizes motor details seems to improve motor imitation. In the current review, four studies reported viewing perspective of the stimuli, which always consisted of frontal perspective (17, 19, 28, 29) with the addition of sagittal perspective in two studies (19, 28). Video-clips delivered to patients were mainly focused on tasks that emphasized body-weight shifting (i.e., step initiation, stepping an obstacle, etc.) along the frontal plane. Similarly, frontal perspective allowed authors to propose an accurate observation of physiological motor strategies during conditions that elicited typical motor signs in patients with PD (i.e., walking through a doorway) (56). Coherently, lateral perspective was adopted when visual stimuli focused on motor phenomena occurring especially along the sagittal plane (i.e., sit to stand) (19, 28).

Growing evidence describes an activity of the MNS during observation of both transitive (meaningful gestures in presence of an object) and intransitive (meaningful gestures in absence of an object) actions (57, 58). However, neurophysiological studies demonstrated higher brain activity during observation of transitive compared to intransitive tasks (59–61). In addition, congruence of transitive observed actions (i.e., grasping) in context has been reported to influence the MNS activity (62, 63). Included studies used both transitive (i.e., stair climbing, walking through a doorway, stepping an obstacle, etc.) and intransitive (body-weight shifting, stepping in different directions, etc.) actions within the same study, making it impossible to compare their efficacy. Some studies proposed a progression in complexity of observed and imitated tasks, starting from simple intransitive actions, followed by transitive challenging daily tasks (28, 29). Finally, being related to rehabilitation addressed to improve disability through approaches focused on patients' motor impairments, the choice of AOT stimuli might depend on motor deficits, which occurred during both intransitive and object-oriented tasks.

When considering transitive actions, MNS revealed an increased resonance during observation of actions related to daily life, promoting the inclusion of functional activities in video-clips (64, 65), as in the studies included in the current review. Moreover, the brain response to observation of actions has been demonstrated to be influenced by personal motor repertoire, revealing greater MNS activity when the observed actions belong to motor expertise of observers (66, 67). In addition, activity of the MNS seems to be modulated not only by the previous acquisition of motor skills (motor repertoire) but also by the visual familiarity with observed actions (visual practice) (68, 69), where the similarity of the observed kinematics with the observer's own kinematics seems to enhance the resonance of motor brain areas (70, 71).

In this scenario, it is reasonable to raise the question whether it is better to deliver AOT stimuli representing healthy subjects or patients with the same pathological conditions of observers. In the studies of this review, where AOT was proposed to patients with PD, stimuli showed actions performed by healthy subjects, except for the study of Jaywant et al. (19) which included also patients with PD. Although the use of video-clips representing patients with the same pathological conditions of observers revealed positive results in terms of MNS recruitment in prosthesis users, no studies have investigated the effects of this stimuli characteristics in patients with neurological disorders (55, 72). However, the use of subjects with the same clinical condition as the observers could be limited due to the difficulty in reproducing the features of pathological movements and the need to overcome their motor impairments through AOT stimuli. In fact, whereas the use of a prosthesis is similar in all patients and represents a definitive clinical condition, subjects with neurological diseases have a huge variety of motor manifestations.

Finally, it was hypothesized that observing one's own actions might influence the AOT efficacy (48), but studies are needed to investigate this issue in subjects with PD.

### Limitations

Some limitations of the current review need to be underlined. First, our findings were based on a small number of RCTs, where the majority included a small number of participants. Therefore, when considering the incidence of PD in the general population, we cannot exclude the fact that the small number of retrieved studies might be affected by a publication bias. Second, the included studies had a wide variability in terms of posology of the treatments (from 7 days to 2 months), stimuli characteristics, and modalities of AOT administration (i.e., with or without imitation), and included outcome measures affected by psychometric limitations. Therefore, the decision to set no restrictions on these features might have influenced our findings. Third, follow-ups were heterogeneous in timing and only two of the seven studies had a long-term assessment, hindering the possibility to draw conclusions on persistence of AOT effects over time. Finally, reporting was poor in some studies, which only reported that there were no significant between-group differences, without reporting the treatment effects.

### Implications for Research and Practice

The review suggests the usefulness of AOT for improving motor function in patients with PD. In particular, treatments lasting at least 4 weeks and incorporating ecological auditory stimuli are reported to induce changes on functional abilities and quality of life. Moreover, imitation of observed actions is suggested to further enhance motor recovery, even though the potential usefulness of AOT alone needs additional investigations. When applied to upper limb, a single session of AOT seems to be enough to reduce bradykinesia, leading us to hypothesize cumulative effects after repeated sessions. In addition, visual stimuli should facilitate patients' empathy through the person-related perspective (third person for locomotor tasks and first person for upper limb activities), the use of transitive actions belonging to patients' motor repertoire, and the similarity of actors with the clinical condition of observers. Meanwhile, the viewing perspective should be taken into account in order to allow patients to focus on movement details.

Future studies with larger number of participants, higher methodological quality, and longer follow-ups are needed to better define the posology of AOT interventions in patients with PD. In addition, the included studies were mainly focused on walking ability or gait-related motor signs whereas additional studies would need to understand AOT effects on other motor and functional domains reported as compromised in these patients. Moreover, AOT alone or in association with other approaches characterized by partial overlap of neural substrates (i.e., motor imagery) deserve further investigations. Finally, future studies should be addressed to study the characteristics of the most effective stimuli.

## Conclusions

In conclusion, AOT leads to improvements in motor and functional performance in patients with PD, especially in terms of walking abilities and gait-related disorders. The characteristics of the training and the visual stimuli delivered to patients play a fundamental role in determining the AOT effects. High-quality randomized controlled trials investigating effects of AOT on less explored motor domains such as postural stability, rate of falls, and functional independence could further expand the applicability of AOT in rehabilitation of patients with PD. Finally, a substantial agreement on the use of AOT stimuli with transitive actions belonging to patients' motor repertoire has been reported. However, original studies aimed at comparing the use of first-person vs. third-person perspective or the observation of video-clips with healthy subjects vs. PD patients as actors could promote additional benefits on recovery induced by AOT.

## Author Contributions

FT and RG contributed to conception, design of the study. FT, PA, and EC conducted the database search and extracted the data. EC implemented the data analysis. FT and RG wrote the original draft, whereas PA and EC contributed to manuscript revision. All authors reviewed and approved the final version of the manuscript.

## Conflict of Interest

The authors declare that the research was conducted in the absence of any commercial or financial relationships that could be construed as a potential conflict of interest.

## Abbreviations

AOT: Action Observation Therapy
PD: Parkinson's disease
MNS: Mirror Neuron System
PRISMA: Preferred Reporting Items for Systematic Reviews and Meta-analysis
H&Y: Hoehn&Yahr
MMSE: Mini-Mental Status Examination
UPDRS: Unified Parkinson's Disease Rating Scale
FoG-diary: Freezing of Gait diary
FoG-Q: Freezing of Gait Questionnaire
PDQ-39: Parkinson's disease Questionnaire -39 items
BBS: Berg Balance Scale
TUG: Timed Up and Go
10 MWT: 10 Meters Walking Test
FIM: Functional Independence Measure
6 MWT: 6 Minutes Walking Test
fMRI: functional Magnetic Resonance Imaging.
