# Supplementary material for: Efficacy and Characteristics of the Stimuli of Action Observation Therapy in Subjects With Parkinson's Disease: A Systematic Review
Source: Front Neurol. 2020 Aug 13;11:808. doi: 10.3389/fneur.2020.00808 (PMC7438447; doi:10.3389/fneur.2020.00808)
Supplement: Supplementary file 1 [file Table_1.DOCX]

Supplementary Material

**Appendix A.** PubMed search strategy

("Parkinson Disease"[Mesh] OR "Parkinson's disease" OR Parkinson* OR PD)

AND

("Observation"[Mesh] OR "Action observation" OR "Action observation training" OR "Action observation treatment" OR "Action observation therapy" OR "Action observation-execution" OR "Motor observation" OR "Movement observation" OR "Motion observation" OR "Gesture observation")

**Appendix B.** Methodological quality of included studies (PEDro score 0-10)

|  | **Eligibility criteria** | **Random allocation** | **Concealed allocation** | **Baseline comparability** | **Participants blinded** | **Clinician blinded** | **Assessor blinded** | **Adequate follow-up** | **Intention-to-treat analysis** | **Between-group analysis** | **Point estimates and variability** | **Total score (0-10)** |
| --- | --- | --- | --- | --- | --- | --- | --- | --- | --- | --- | --- | --- |
| Agosta et al. 2017 | yes | 1 | 1 | 1 | 0 | 0 | 1 | 1 | 0 | 1 | 1 | **7** |
| Buccino et al. 2011 | yes | 1 | 0 | 1 | 0 | 0 | 1 | 0 | 0 | 1 | 0 | **4** |
| Jaywant at al. 2016 | yes | 1 | 1 | 1 | 0 | 0 | 0 | 1 | 1 | 1 | 1 | **7** |
| Mezzarobba et al. 2018 | yes | 1 | 1 | 1 | 0 | 0 | 1 | 1 | 1 | 1 | 1 | **8** |
| Pelosin et al. 2010 | yes | 1 | 0 | 1 | 0 | 0 | 1 | 1 | 1 | 1 | 1 | **7** |
| Pelosin et al. 2013 | yes | 1 | 0 | 1 | 0 | 0 | 1 | 0 | 0 | 1 | 1 | **5** |
| Pelosin et al. 2018 | yes | 1 | 0 | 1 | 0 | 0 | 0 | 1 | 0 | 1 | 1 | **5** |

**
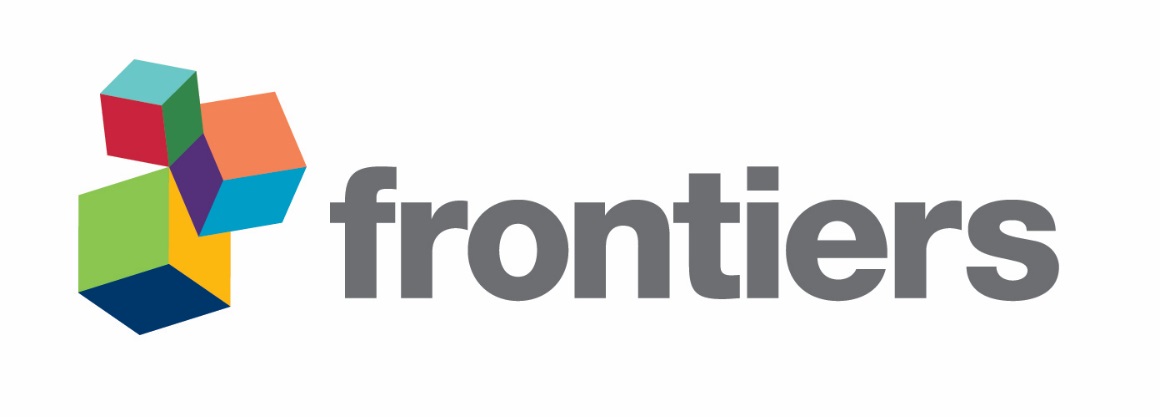
**
